# A Group of Tumor-Suppressive micro-RNAs Changes Expression Coordinately in Colon Cancer

**DOI:** 10.3390/cimb45020063

**Published:** 2023-01-20

**Authors:** Ovidiu Farc, Liviuta Budisan, Ioana Berindan-Neagoe, Cornelia Braicu, Oana Zanoaga, Florin Zaharie, Victor Cristea

**Affiliations:** 1 Immunology Department, “Iuliu Hatieganu” University of Medicine and Pharmacy, 400347 Cluj-Napoca, Romania; 2 Research Center for Functional Genomics, Biomedicine and Translational Medicine “Iuliu Hatieganu” University of Medicine and Pharmacy, 400347 Cluj-Napoca, Romania; 3 Surgical Department, “Iuliu Hatieganu” University of Medicine and Pharmacy, 400347 Cluj-Napoca, Romania

**Keywords:** micro-RNA, network, correlations, transcription factor

## Abstract

MicroRNAs (miRNAs) are molecules with a role in the post-transcriptional regulation of messenger RNA, being involved in a wide range of biological and pathological processes. In the present study, we aim to characterize the behavior of a few miRNAs with roles in the cell cycle and differentiation of colon cancer (CC) cells. The present work considers miRNAs as reflections of the complex cellular processes in which they are generated, their observed variations being used to characterize the molecular networks in which they are part and through which cell proliferation is achieved. Tumoral and adjacent normal tissue samples were obtained from 40 CC patients, and the expression of miR-29a, miR-146a, miR-215 and miR-449 were determined by qRT-PCR analysis. Subsequent bioinformatic analysis was performed to highlight the transcription factors (TFs) network that regulate the miRNAs and functionally characterizes this network. There was a significant decrease in the expression of all miRNAs in tumor tissue. All miRNAs were positively correlated with each other. The analysis of the TF network showed tightly connected functional modules related to the cell cycle and associated processes. The four miRNAs are downregulated in CC; they are strongly correlated, showing coherence within the cellular network that regulates them and highlighting possible approach strategies.

## 1. Introduction

Colorectal cancer (CRC) is a disease with a global increase in incidence, being currently the third most diagnosed and the second cause of death among cancers worldwide [1]. It remains a major public health problem, in spite of the numerous efforts that were made to improve diagnosis and therapy [2]. Genomic alterations, as well as inflammatory and microenvironmental modifications, are involved in the pathogenesis of this disease [3,4].

In this context, recent research uncovered a growing role of what was called the “dark matter” of the genome, the non-coding part of the cellular DNA, which, far from being silent DNA, as it has been believed to be, plays crucial roles in the coordination of gene expression and cellular processes. This non-coding DNA exerts its functions by the transcription of different non-coding RNAs, such as long non-coding RNAs (lncRNAs), circular RNAs (circRNAs), microRNAs (miRNAs) and others [5].

MicroRNAs (miRNAs) are short (15–29 bases), single-stranded RNAs whose main role is the posttranscriptional regulation of the messenger RNA [6]. They represent a mechanism ubiquitously present in cell biology, in multiple processes such as the fine-tuning of protein production by ribosomes, cell cycle, differentiation of certain cell types or the development of certain body regions. In the last period, the implications of miRNAs in the pathogenesis of malignant tumors are increasingly recognized and studied. It has been shown that some of these molecules change their expression in the tumor tissues, exerting stimulatory or inhibitory influences on tumor growth [7].

The present study aims to investigate the behavior of a few miRNAs in the tumoral tissue in colon cancer (CC) and study potential applications. The selected miRNAs exert important roles in the cell cycle and differentiation of the intestinal epithelium, being involved in the biology of the colon normal and tumor cells.

MicroRNA-29a (miR-29a) is located on chromosome 7q 32.3, on the exon 2 of a long non-coding RNA (*LOC646329*), with roles in cell proliferation [8]. It is synthesized under the transcriptional control of NfkB (Nuclear factor kappa B), Myc and TCF/LEF (T-cell factor/lymphoid enhancer factor), among others [9,10]. Its targets are extracellular matrix regulators [10], molecules from the cell proliferation and differentiation pathways or DNA methylation [11]. Mir-29a was associated with a good prognosis in CRC [12], there is also a study that showed a tumor-promoting role of this miRNA [13]

MiR-146a is located on chromosome 5q 33.3 on the gene of a lncRNA (*MIR3124HG*); it is activated by NfkB, CEBPα (CCAAT-enhancer binding protein α) and TGFβ (Transforming growth factor β) and inhibited by Myc [14]. It ensures the negative regulation of immune pathways and it also regulates cell proliferation, adhesion, migration [14] and cell differentiation (by inhibiting NUMB-a Notch inhibitor, thus leading to the proliferation of intestinal progenitor cells] [15]. The effect on colorectal tumors is generally inhibitory [16], although there are reports of a prometastatic [17] or immunosuppressive [18] role. The evidence points towards a pleiotropic, mostly antitumoral role [19].

MiR-215 is an intronic miRNA located on chromosome 1q 41.1, on the gene of isoleucyl-tRNA synthetase 2, on the fragile site FRA1A, deleted in many cancers [20]. It is generated under the transcriptional control of p53 protein but also of some lineage-specific transcription factors [hepatic-specific factor HNF-1 or intestinal CDX-1] [21] or Hif1α in hypoxia [20]. It targets factors from the proliferative pathways, the cell cycle, apoptosis [22], adhesion and migration or differentiation [20,21]. In colorectal cancer it has a tumor-suppressive role and it decreases early in the evolution of tumors, and it arrests the cell cycle in a p53-dependent manner [23].

MiR-449 is situated on chromosome 5, in a cluster with miR-34 and it is also an intronic miRNA on the second intron of the *CDC20B* gene; it is positively regulated at the transcriptional level, by the E2F1 factor [24] and also by cell type-specific transcription factors (TFs), such as SOX17 and FOXJ1 for the ciliary differentiation [25]. It represents negative feedback in the case of replicative stress by increased E2F1, which might otherwise lead to apoptosis [24]. Its known targets are at the level of the cell cycle, cell differentiation, epigenetic control of DNA expression and cell adhesion, among others [25]. In CRC, it has been found to be decreased; the inhibition of mir 446a led to increased proliferation [26]. It decreases through hypermethylation [25].

MicroRNAs are part of the complex molecular circuitry of signaling and transcriptional networks that are activated in cell biology and cell division, in which they accomplish different inhibitory or stimulatory roles concerning cell cycle, differentiation, cell migration or angiogenesis [27,28]. To have an accurate perspective on the role of these molecules, they should be considered in their biological context, acting as parts of these complex networks; such a perspective may open the way for strategic approaches, which, rather than using or inhibiting a single molecule [29], aim at targeting entire networks or molecular ensembles. One of our starting hypotheses was that there is coherence and coordination in the events at the signaling and transcriptional level, through which cell cycle and cell proliferation are accomplished. We expected, by consequence, to find a system of correlations between the factors involved, including miRNAs, that reflects this and that also permits strategic approaches.

## 2. Materials and Methods

The present study is a prospective study in which 40 patients with colon cancer were enrolled (Table 1). The patients were treated in the surgical clinics of the Regional Institute of Gastroenterology and Hepatology from Cluj-Napoca and of the County Emergency Hospital of Sibiu, Romania, between November 2018 and March 2020.

The study included patients with colon adenocarcinoma confirmed by histopathological examination. The following patients were excluded from the study: patients that had undergone treatments that could alter miRNA expression in tissue (chemotherapy, radiotherapy, others).

The study was approved by the Ethics Committee of the Regional Institute of Gastroenterology and Hepatology from Cluj-Napoca, Romania (approval no. 2769/1.03.2018, of the Iuliu Hatieganu Medicine and Pharmacy University from Cluj-Napoca, Romania (approval no. 40/02.04.2018), and of the County Emergency Hospital of Sibiu, Romania (approval no. 10759/23 05.2019).

*Collection and storage of the samples.* Colon tumor tissue and adjacent normal tissue were obtained from the 40 patients through intraoperative section. The samples were prepared and stored at −170 °C in liquid nitrogen.

*miRNA extraction and quality control.* Total RNA from tissue was extracted using TriReagent (Thermo Fischer Scientific, Waltham, MA, USA) based on the producer’s recommended protocol. RNA concentrations and quality were evaluated using NanoDrop-1100. The sample purity was evaluated based on the spectral data and purity ratios.

*miRNAs and housekeeping gene sequences* (from miRbase):

 hsa-miR-29a-ACUGAUUUCUUUUGGUGUUCAG
 hsa-miR-146a-UGAGAACUGAAUUCCAUGGGUU
 hsa-miR-215-AUGACCUAUGAAUUGACAGAC
 hsa-miR-449-UGGCAGUGUAUUGUUAGCUGGU
RNU6B-CGCAAGGATGACACGCAAATTCGTGAAGCGTTCCATATTTTT
RNU48-GATGACCCCAGGTAACTCTGAGTGTGTCGCTGATGCCATCAC-
CGCAGCGCTCTGACC

q*RT-PCR* (real-time quantitative reverse transcription polymerase chain reaction) analysis. qRT-PCR miRNA primer assays were provided from Thermo Fischer Scientific (Waltham, MA, USA), as follows: for miR-29a, cat. No. 002447; for miR-146a, cat. No. 002163; for miR-215, cat. No. 000518; finally, for miR-449, we used cat. No. 001030. Assays with ID no. 001093 for RNU6B and 001006 for RNU48 were used as housekeeping miRNAs.

A total of 50 ng RNA for miRNA (miR-29a-5p, miR-146a-5p, miR 215-5p, miR 449-5p) expression was transcribed into cDNA using a TaqMan MicroRNA Reverse Transcription Kit (Applied Biosystems, Thermo Fischer Scientific, Waltham, MA, USA). For miRNA amplification, we used a TaqMan Fast Advanced Master Mix (Applied Biosystems, Thermo Fischer Scientific, Waltham, MA, USA). Relative expression levels of miRNAs were calculated using ΔΔCt method and for the graphical representation GraphPad Prism 6 (GraphPad software inc., San Diego, CA, USA) was used. qRT-PCR was performed with a ViiA™ 7 System (Thermo Fischer Scientific, Waltham, MA, USA) in a 5 µL volume using a 384-well plate. All the samples were evaluated in duplicate (40 samples of tumoral tissue and 40 samples of adjacent normal tissue, each one in duplicate, for each miRNA).

*Statistical analysis.* The analysis of the statistical significance of the difference between tissue miRNA levels in normal and tumoral tissues, as well as between tumor locations was performed with the Mann–Whitney U-test for non-normally distributed data. The analysis of the tissue levels of miRNAs in different stages and tumor grades was performed using Kruskal–Wallis test for data with non-normal distribution. Post-hoc analysis was performed using Dunn’s test. Data normality was tested using the Anderson-Darling test. Since there were no paired variables showing bivariate normality, the correlation matrices were generated using the Spearman correlation test.

Graphical representation of tissue expression levels of miRNAs was performed using GraphPad Prism 6 (GraphPad software inc, San Diego, CA, USA). All the other statistical analyzes were performed using XLstat software, version 2021.3.1 (from Addinsoft, New York, NY, USA) [30]. *p* < 0.05 was considered statistically significant.

To identify the transcriptional factors on which the expression of miRNAs depends, the online instrument TransMir (version 2.0) was used [31]. The correlation network between these transcription factors was built using GeneMania software [32] and GeneMania Cytoscape plug-in [33], and the functional analysis of this network was carried out by using Cytoscape (version 3.9.1) [33], the Cytoscape plug-ins EnrichmentMap [34] and Reactome FI [35]; the correlations between the cellular functions were confirmed by using the Auto-Annotate plug-in from Cytoscape [33], and also by visually identifying correlations between modules in the network.

## 3. Results

The biological background and the clinico-pathological features of patients are presented in Table 1.

**Figure 1:**
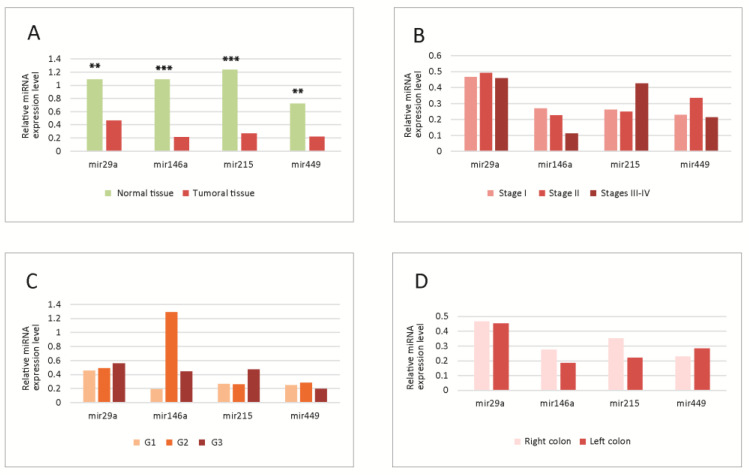
(**A**) Tissue levels of miRNAs in normal versus tumoral tissue. Significant differences were observed between normal and tumoral tissues; all four miRNAs were downregulated in tumoral tissue; (**B**–**D**) Tissue levels of miRNAs in different tumor TNM stages (**B**), WHO grades (**C**) and locations (**D**); no statistically significant differences were observed between the levels in different stages, grades or locations. G1-G3-tumor WHO grade. MiRNA level is expressed as fold change (FC). ** *p* < 0.01; *** *p* < 0.001. WHO-World Health Organization.

*MiRNA tissue levels.* The levels of miRNAs in normal versus tumoral tissue, as well as in different tumor stages, grades and locations are summarized in Figure 1.

The levels were significantly lower for all molecules in tumoral versus normal tissue;, the most significantly downregulated was miR-215 (Figure 1A).

No significant differences were observed in the analysis in different tumor stages (Figure 1B), grades (Figure 1C) or locations (Figure 1D). However, some trends were observed, miR-29a and miR-146a decreased slightly, and miR-215 increased towards the advanced stages; miR-146a had higher levels in G2, while miR-29a and miR-215 were increased in G3 tumors; concerning the location, there was a slight decrease in miR-215 and 146a in the left colon tumors.

**Figure 2:**
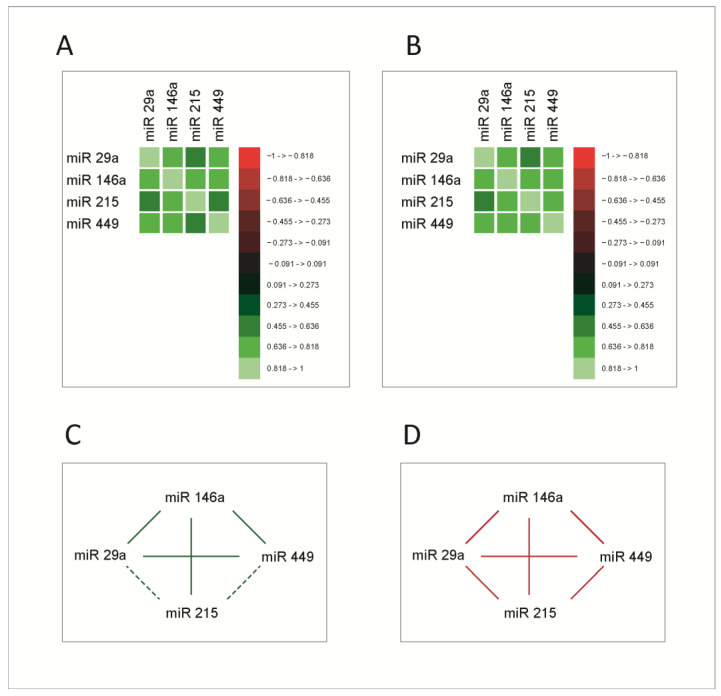
Correlation matrices between microRNAs in normal (**A**) and tumoral (**B**) tissue; the values on the color scale represent the Spearman ρ coefficient. (**C**,**D**) Correlation network in normal and tumoral tissue, respectively. Continuous line- correlations with Spearman ρ coefficient ≥0.5; dashed line-significant correlations with Spearman ρ coefficient < 0.5; green line-correlations in healthy tissue; red line-correlations in the tumoral tissue. The molecules had significant correlations between them, stronger in the tumoral tissue.

As mentioned, miRNAs exert their roles in the context of complex signaling and transcriptional networks, through which the main cellular functions are performed. As a consequence, we asked whether increases or decreases in miRNA expression levels occur in a coordinated manner, reflecting eventually the variations of the function in which they are generated and to which they contribute, in this case, the cell cycle. To test this hypothesis, we studied correlations between the four miRNAs. The correlation analysis was performed using Spearman correlation coefficients. Correlation matrices are presented in Figure 2A,B, for both normal and tumoral tissue; the correlation network between miRNAs in healthy and tumoral tissue is presented in Figure 2C,D.

All molecules exhibited significant correlations with each other; miR-215 had stronger correlations with miR-29a and miR-449 in tumoral tissue. This supports the hypothesis that these molecules are functionally connected and prompts a more extensive analysis of these connections. Given the fact that miRNAs act mainly by the post-transcriptional inhibition of RNA messages, this mechanism does not provide an explanation for such strong and intricate correlations between them. Consequently, an explanation for this correlated behavior was sought within the biological mechanisms that regulate the expression of the four miRNAs.

**Figure 3:**
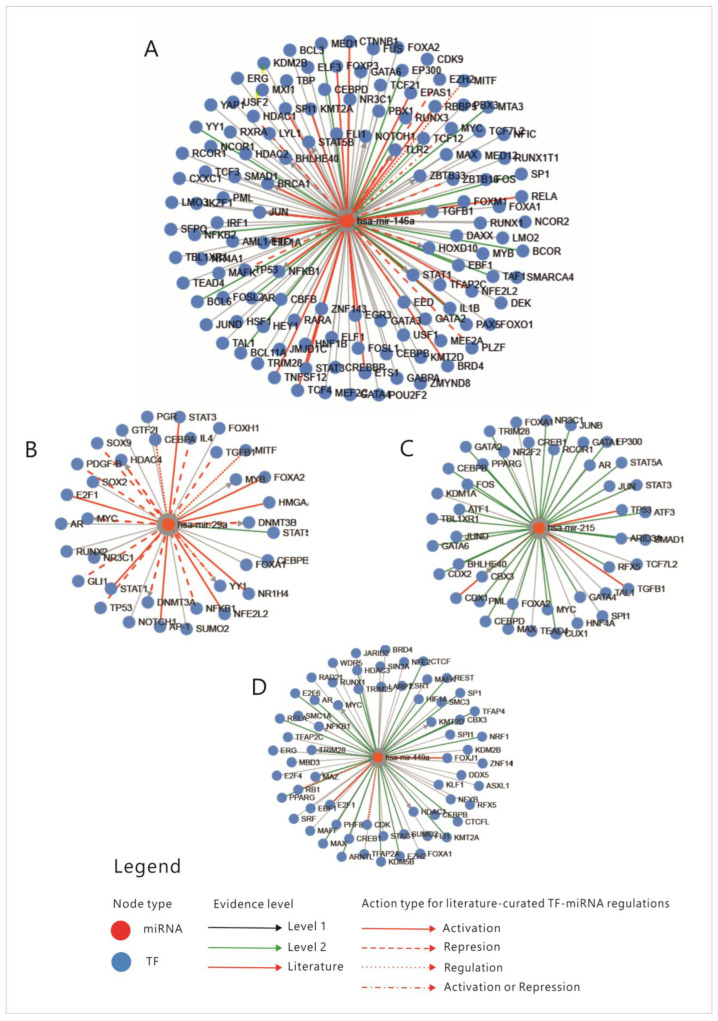
Transcription factors that regulate the four miRNAs. (**A**) miR-146A; (**B**) miR-29A; (**C**) miR-215; (**D**) miR-449. MiR-micro-RNA; Levels 1,2- FT-miRNA regulations derived from ChIP sequencing; level 2 promoter-supported by high-throughput experimental data; TF-transcription factor; mi-RNA-micro-RNA; (TransMir, v.2.0) [31].

First, an analysis of the four miRNAs regulation through transcription factors (TFs) was performed; the relations between miRNAs and TFs that regulate each one of them are presented in Figure 3. Enrichment analysis of miRNAs-TFs regulation is shown in Table S1.

**Figure 4:**
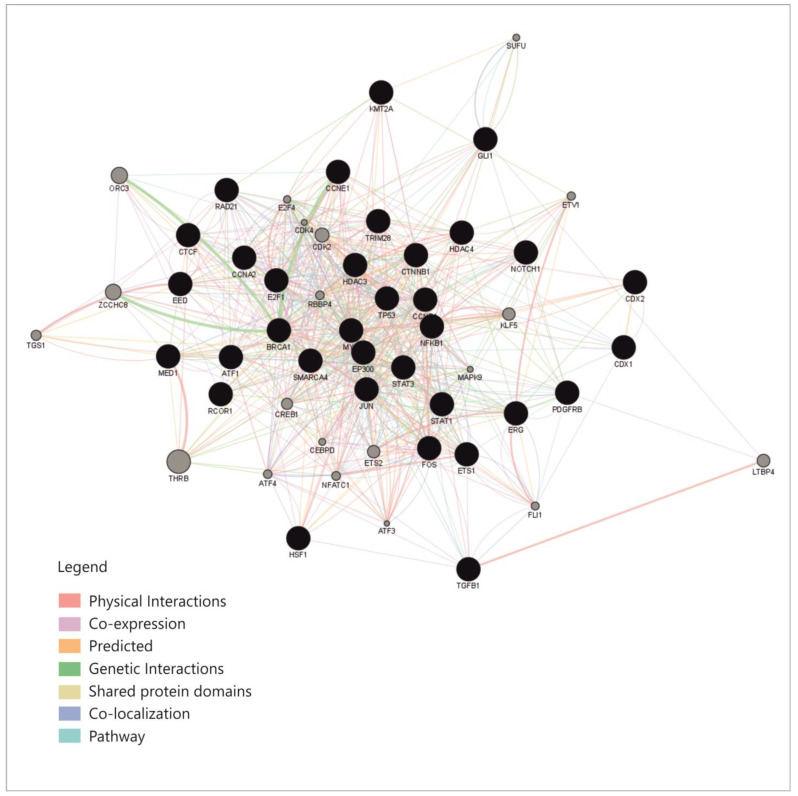
The correlation network between the transciption factors that regulate the four miRNAs (GeneMania and Cytoscape GeneMania plug-in) [32,33].

An overview of these transcription factors shows that they represent pathways that regulate proliferation (JAK-STAT, TGFβ, WNT, NOTCH-1), cell differentiation (SOX9, CDX 1-2, RUNX2), some of them belong to the group of immediate-early genes (such as FOS, MYC, JUN or CREB-1), while others are cell cycle regulators such as TP53, E2F1-6 or RAD21. Some of these TFs regulate basic transcription or chromatin organization, while few of them control cell functions and pathways such as metabolism or inflammation. To explain the connexions between miRNAs, we constructed a correlation network between the TFs that were shown to regulate them. From the aforementioned enrichment analysis, only TFs with fold change (FC) greater than 1.5 were considered (Table S1); TFs that direct differentiation in the non-intestinal tissues, such as HNF4, SOX9 or GATA3, were also excluded from the analysis. The correlation network was built based on the following: physical interaction between molecules, co-expression, predicted and genetic interactions, shared protein domains, co-localization and pathway sharing. The diagram showing correlations between the TFs is presented in Figure 4.

This network shows relatively high connectivity, with a density of 0.363, an average of 19.22 neighbors for each node and a clustering coefficient of 0.518 [33]; this already provides an explanation for miRNAs correlations, which appear to be caused by correlations within the network of TFs that regulate them.

This, however, does not explain how the studied miRNAs are regulated in the context of the main cellular processes and functions. To clarify this aspect, we aimed to determine in which cellular functions and pathways the TFs from the mentioned network are integrated and the way in which these pathways and functions interact.

**Figure 5:**
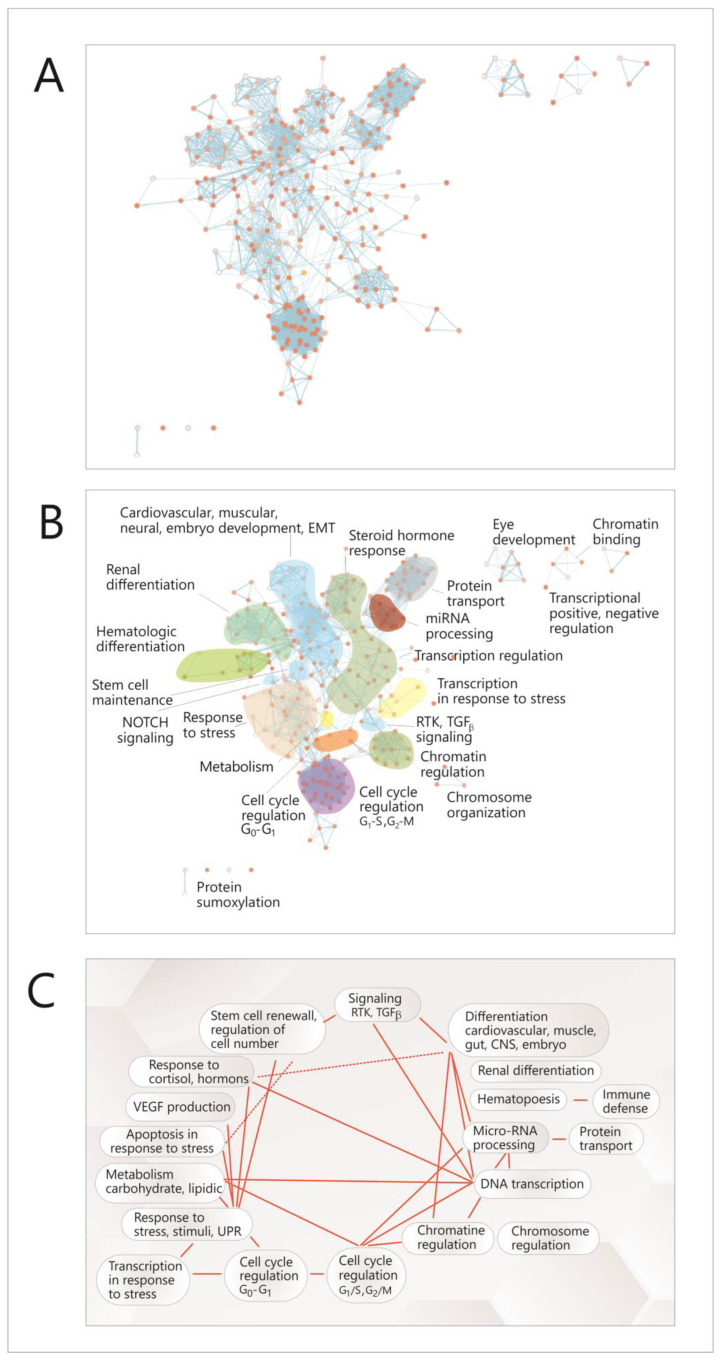
Functional analysis of the correlation network between the TFs that control the expression of the miRNAs. (**A**) Network with complex nodes (Cytoscape with EnrichmentMap plug-in) [33,34], Red dots-network nodes; Blue lines-network edges. (**B**) The diagram highlights functional modules in the network (Cytoscape with Reactome FI plug-in) [35]: (**C**) correlations between the network modules (Cytoscape with AutoAnnotate plug-in) [33]. TF-transcription factor; EMT-epithelial-to-mesenchimal transition; G1,S,G2,M-stages in cell cycle; UPR- unfolded protein response; RTK-receptor-thyrosin-kinase.

To achieve this objective, a functional analysis of this network was performed by searching functional modules within it, based on the GO (Gene Ontology) annotation system and the reactome FI algorithm [35] and analyzing the correlations between the resulting modules. Due to the multi-level architecture of functions within the genome, the correlations between TFs were not directly translated into higher-level modules representing cell functions, but instead, the GO pathways in which TFs are present were identified first; then, a new network was built, with complex nodes, each one of them consisting of such a pathway (Figure 5A, Figure S1); these nodes assemble to form higher hierarchy clusters or modules, representing main cellular functions and processes (Figure 5B); fourteen such modules were identified (Figure 5B); eight of which are connected by both intra-and inter-module links (Figure 5A–C). In Figure S1, each of these nodes is shown, and in Table S2, all the functional modules are presented, with their node list and the genes (TFs) that are present in nodes.

Most of these modules are related to the cell cycle and differentiation (chromatin and chromosome regulation, transcription regulation, miRNA biogenesis, different types of differentiation or signaling pathways); there are also some modules representing other cellular functions such as response to stress, hormone response, metabolism, apoptosis in response to stress or transcription in response to stress. It can be seen that there are correlations between these modules (Figure 5A,C), and within each module (Figure 5A); the richest intra-module correlation network is present in the modules corresponding to the cell cycle (G1-G2, G2-S), miRNA regulation, protein transport and cardiovascular development. Overall, the network had a density of 0.057, an average number of neighbors of 15,827 for a node and a clustering coefficient of 0.687 [33].

Together, these data show a high level of coherence and interdependence in the processes concerning cell proliferation, in the meantime providing an explanation for the correlated behavior of the four studied miRNAs in the context of these cellular functions.

**Figure 6:**
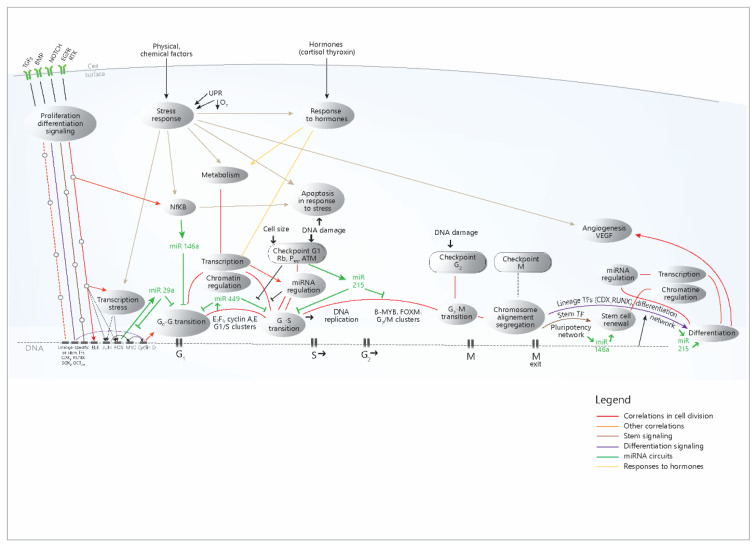
Proposed model concerning the cellular networks and pathways that control the expression of the four miRNAs. It can be seen that in the case of cell cycle-related processes, there is a stream of events that begins with the signaling pathways activation, then the immediate-early genes are activated, then the transition G0-G1 occurs, followed by the transition G1-S and G2-M, and finally, the mitotic exit and the differentiation of the daughter cells take place. The successive transcriptional waves are coordinated by specific master TFs for each one of them, but each step is accomplished through the action on the chromatin, which makes possible the basic transcription; after that, the post-transcriptional regulation through miRNAs occurs (all these processes were shown to be correlated in our analysis). The differentiation is signaled from the start through the presented pathways, but it occurs only at the end of the cell cycle (when also signaling events may occur). It has also been shown to correlate with these basic chromatin, transcription processes and regulation through miRNAs. The other processes (response to stimuli, response to hormones, transcription in response to stress) occur independently, (in response to their own stimuli), but in networks that communicate with the cell cycle. Red, light brown or yellow lines- correlations from the present study; green line- miRNA circuits; UPR-Unfolded protein response (Endoplasmic reticulum stress); TF-transcription factors; G1,S,G2,M- stages in the cell cycle. The model was built based on the conclusion of the present study and also on other literature data [10,14,20,24,36,37].

To put these data in their cellular context and causal relationship, a model is presented, which summarizes the data and correlations from the present study (Figure 6).

The diagram shows the role of the four miRNAs in the signaling and transcriptional networks that accomplish cell proliferation and other cellular processes; miR-29a and miR-449 are mainly synthesized as a consequence of the activation of proliferation circuits and exert inhibitory roles in these circuits, most likely as negative feedback loops. miR-215 increases in the DNA damage response and inhibits the cell cycle; the anti-inflammatory role of miR-146a mainly refers to its action in immunocytes (but not only), while in the intestinal epithelium, it mostly inhibits the cell cycle. MiR-215 and miR-146a are also present at the end of the cell cycle, with the opposite role: the former stimulates differentiation, while the latter contributes to stem cell renewal. The diagram simplifies much of the complex mechanisms that trigger the action of the four miRNAs, which were shown in Figure 3; however, the multitude of TFs that regulate the miRNAs are mainly distributed in these proliferation pathways, as the present study has shown. Overall, the diagram explains the concerted modifications in miRNA levels through the coherence of the cellular mechanisms that regulate their expression.

## 4. Discussion

MicroRNAs were intensively studied in colorectal cancer, with regard both to their contribution to the pathogenesis of the disease [38] and to their potential diagnostic or therapeutic implications in CC [39,40].

In the present study, we aimed to characterize the behavior of a few tumor-suppressive miRNAs in colon cancer, find explanations for this behavior and study potential applications. The miRNAs were chosen to represent specific stages in the cell cycle and cell differentiation of the colon normal and tumor cell. The level of miR-29a and 146a vas found to decrease in the tumoral tissue; other studies have found increased levels of miR-29a [41] or miR-146a [18,41]; decreased levels of these two miRNAs have also been found [42,43]. An explanation of this behavior could be given considering the activation mechanisms of these miRNAs; miR-29a is subject to a complex transcriptional regulation through multiple TFs, mostly subsequent to the proliferation pathways; however, some of these factors increase, while other decrease miR-29a level, giving to this miRNA the possibility to have a pleiotropic role in cancer [8,9,10]; miR-146a, besides being antiinflammatory, is subject to the same complex regulation, with the same result- a pleiotropic, context-dependent behavior [14]. By contrast, both miR-215 and miR-449 are activated in differentiation or in the DNA damage response [20,24,25], which are dysregulated in cancer; as a consequence, a decrease in their levels in CC would be expected. Indeed, miR-215 and miR-449 have constantly been found to decrease in the tumor tissue of CC compared to normal [22,23,26], similar to the findings of the present study. Epigenetic silencing is another mechanism that may account for the global decrease in the levels of these miRNAs [20,26].

The highly significant differences in miRNA expression between normal and tumoral tissues contrast with the lack of significant differences between tumor stages, grades or locations. This suggests a relative homogeneity of mechanisms in all tumors, regardless of their stage, grade or location. The correlation system between miRNAs, which is relatively similar in tumors compared to normal tissue, suggests a similarity of mechanisms that control miRNAs in the two situations, leading, however, to an overall decreased miRNAs level in tumors.

Network approaches have become increasingly frequent in miRNA study, given the fact that miRNAs act in complex molecular networks and environments, and these approaches may provide a more accurate understanding of their biological functions [27,28].

As mentioned, the four miRNAs were selected to represent certain stages of the cell cycle [10,14,15,20,24], their behavior reflecting in fact the stage in which they are generated. In this regard, one of our starting hypotheses was that during the cell cycle, the signaling, transcriptional and proteic networks operate with a high degree of coherence and coordination, building a system that functions coherently from start to end and where no errors are allowed. Therefore, we expected coherence from its different parts, including miRNAs. Correlation analysis indeed showed a relatively high degree of correlation of the four miRNAs, both in normal and tumoral tissue, stronger in tumors (Figure 2).

Given the lack of known direct biological relations between miRNAs, an explanation for their behavior had to consider the mechanisms that regulate them. A network with a high level of coherence was found, which covers most of the cell cycle and related functions, such as signaling pathways, chromatin or transcription regulation, metabolism and certain differentiation processes, as well as other cellular functions, such as stress or hormone responses. Thus, it was shown that miRNAs correlations indeed reflect the correlations and coherence within their cellular network of origin.

Analyzing network data in the light of the current knowledge about cell biology processes [10,14,20,24,36,37], we propose a model where the tightly correlated data are put in their cellular context (Figure 6), highlighting a flow of events that start from the growth signals and ends with daughter cells differentiation, explaining once more the correlation and coherence of these processes, which in fact arise from their precise genomic, epigenomic and other types of coordination and programming [36,37].

The presented model explains the tight correlations that were seen for the processes in the network, but also explains the biological context and causal sequence of the processes. It also explains the coordinated increase in the four miRNAs, given their position in the network (Figure 6). The diagram also shows a certain modularity of these pathways, with certain sections of a pathway being used by other pathways.

Thus, the presented data are consistent with a view of the cell cycle as a cellular function with a high degree of coherence and interdependence of parts, consisting of an unidirectional flow that, once started, goes forward, identically each time, until its end, with a few differentiation variants. Such a perspective is worth considering, in our opinion, since a correlated system may imply the possibility of coherent strategic approaches.

In this regard, at the conceptual level, the cell cycle may be assimilated to an autocratic network, where the activation of a single upstream point may lead to the activation of the whole subsequent flow of events, which allows its aberrant activation in cancer due to the action of oncogenic stimuli. At the therapeutical level, it may highlight possible inhibition strategies that efficiently approach key points in this flow, with consequences on the whole ensemble. Other strategies may consider interfering with this flow by using other signaling pathways that were shown to influence it, such as cadherin [44], integrin [45], interleukin [46] or differentiation signaling [47].

The vast correlational network of cancer cell biology includes many more elements such as miRNAs, transcription factors, proteins, enzymes, organelles and so on; it may be expected that this system or parts of it might also have a coordinated behavior, allowing to be strategically approached as whole networks.

The present work considers only patients suffering from colon cancer, due to the fact that a previous study performed by our group showed different miRNA profiles in the colon compared to rectal tumors [48]. The main take-home point is that although addressing individual elements (miRNAs, genes, enzymes) for stimulation or inhibition often proved successful, the genome is a complex mechanism that functions as a whole and with coordination between elements, and it should be approached accordingly.

## 5. Conclusions

1. The four analyzed miRNAs exhibit a decreased level in tumors compared to normal tissue;
2. There are strong correlations between them in both tumor and normal tissue;
3. These correlations are explained in the context of the cellular networks in which miRNAs are part, which has been identified as the mechanisms that regulate cell proliferation and differentiation; the subsequent analysis of these networks highlighted a high level of connectivity and internal coherence of these processes;
4. The study has also highlighted the broad communications and interdependencies in cellular and molecular networks during cell proliferation and not only, since analyzing the connexions of a small group of miRNAs implied to consider such a vast array of cellular functions;
5. The study ultimately tests the coherence within the biological system of the intestinal cell, both normal and tumoral. This may have possible therapeutic consequences, since a correlated system may be subject to coherent strategic approaches.

The present study adds to the impressive body of research that highlighted the complexity and coordination of cellular functions in both normal and tumor cells, pointing to an integrated approach to the study and therapy of cancer, which considers the ensemble of the cellular networks of proteins, mediators and nucleic acids, looking for solutions into this complex edifice. The study of this intricate cellular network is a worthwhile endeavor in the search for solutions to the great problem that cancer still poses to the scientific world.

## Figures and Tables

**Table 1:** The clinico-pathological characteristics of patients.

1. Mean age (years)		67.9 (11.52) ^1^
2. Sex	Male	18 (45%)
	Female	22 (55%)
3. Tumor histology	Adenocarcinoma	40 (100%)
4. Tumor TNM stage	1	5 (12.5%)
	2	16 (40%)
	3	14 (35%)
	4	5 (12.5%)
5. Tumor WHO grade	1	9 (22.5%)
	2	24 (60%)
	3	7 (17.5%)
6. Tumor location	Right colon	21 (52.5%)
	Left colon	19 (47.5%)
7. Comorbid conditions	Inflammatory conditions (RA, IBD, LE)	0 (0%)
	Diabetes mellitus	5 (12.5%)
	Obesity	2 (5%)
	Cirrhosis	1 (2.5%)
	Ascites	1 (2.5%)
	CKD	1 (2.5%)
	Chronic bronchitis	1 (2.5%)

^1^-Mean (standard deviation); WHO-World Health Organization; RA-rheumatoid arthritis; IBD-inflammatory bowel disease; LE-lupus erythematosus; CKD-chronic kidney disease.

## Data Availability

The data that support the conclusion of this study will be made available from the authors on reasonable request.

## Supplementary Materials

The following supporting information can be downloaded at: https://www.mdpi.com/article/10.3390/cimb45020063/s1, Figure S1: Network with annotations; Table S1: Enrichment analysis of the miRNA-transcription factors relation; Table S2: Network modules.
