# Supplementary material for: A Group of Tumor-Suppressive micro-RNAs Changes Expression Coordinately in Colon Cancer
Source: Curr Issues Mol Biol. 2023 Jan 20;45(2):975–89. doi: 10.3390/cimb45020063 (PMC9955927; doi:10.3390/cimb45020063)
Supplement: Supplementary file 1 [file cimb-45-00063-s001.zip › Supplementary Figure S1 with legend.pdf]

Figure S1. Network with annotated nodes

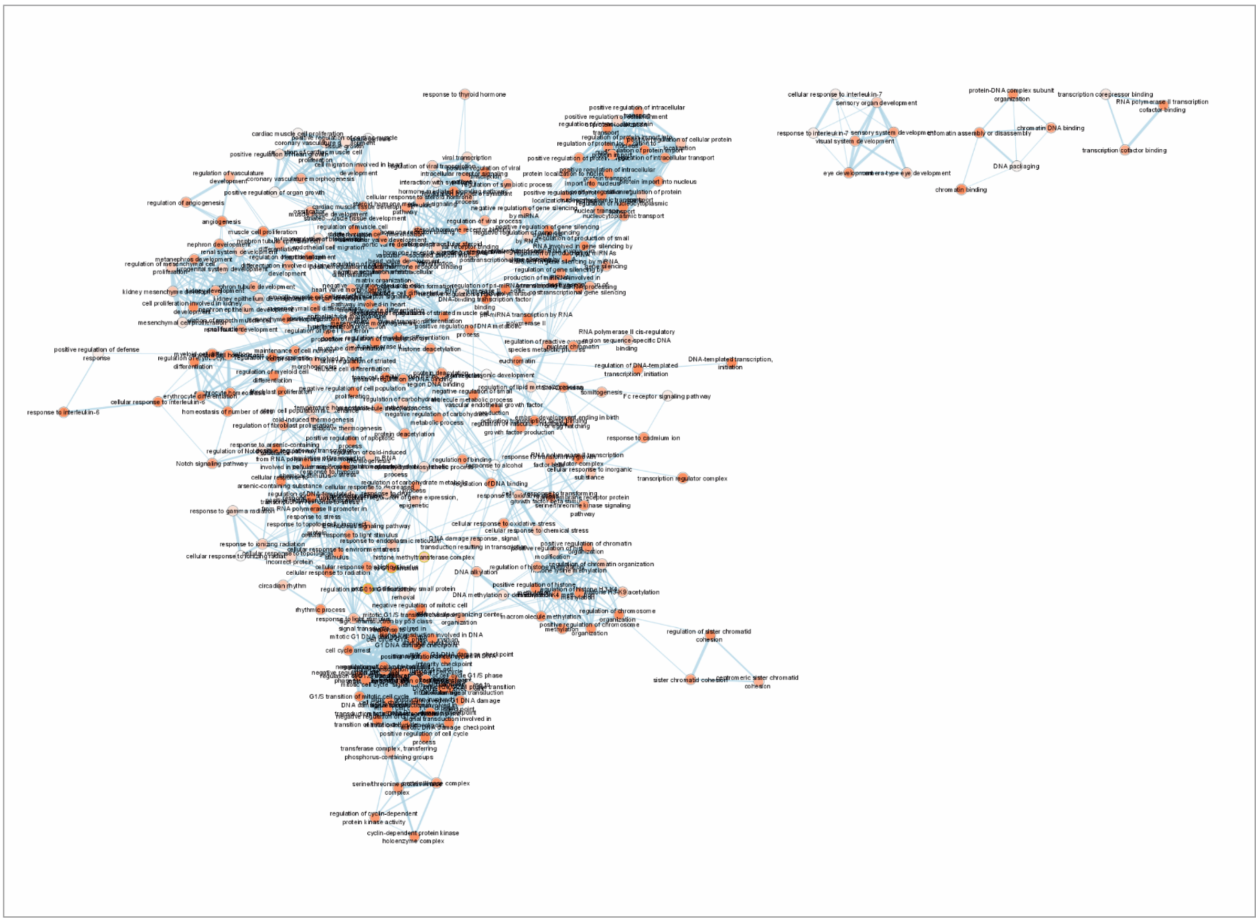

Figure S1-Network with annotations. The GO (Gene Ontology) annotation of each node of the network in Figure 5A is presented. Red dots-nodes in network; Blue lines-edges in network; labels-GO annotation terms.
