# Supplementary material for: A Group of Tumor-Suppressive micro-RNAs Changes Expression Coordinately in Colon Cancer
Source: Curr Issues Mol Biol. 2023 Jan 20;45(2):975–89. doi: 10.3390/cimb45020063 (PMC9955927; doi:10.3390/cimb45020063)
Supplement: Supplementary file 1 [file cimb-45-00063-s001.zip › Supplementary Table S1-Enrichment TF-miRNA.pdf]

Table S1- Enrichment analysis for transcription factors that regulate the expression of the four miRNAs [31]

Nr	Category	Term		percent	Fold change	*p*-value	Bonferroni
1	Transcription factor	AP-1	1	0.07692308	25.46153846	0.04155275	1
2	Transcription factor	AR	2	0.00485437	1.60679612	0.36902917	1
3	Transcription factor	ARID3A	1	0.08333333	27.58333333	0.03862842	1
4	Transcription factor	ARNTL	1	0.00364964	1.2080292	0.6051722	1
5	Transcription factor	ATF1	1	0.01162791	3.84883721	0.23764541	1
6	Transcription factor	ATF3	1	0.00653595	2.16339869	0.38958919	1
7	Transcription factor	BCL11A	1	0.16666667	55.16666667	0.0209417	1
8	Transcription factor	BCL6	1	0.00909091	3.00909091	0.2949974	1
9	Transcription factor	BCOR	1	0.00662252	2.19205298	0.38541367	1
10	Transcription factor	BHLHE40	1	0.00617284	2.04320988	0.40811634	1
11	Transcription factor	BRCA1	1	0.01333333	4.41333333	0.2102208	1
12	Transcription factor	BRD4	1	0.00271739	0.89945652	0.72852769	1
13	Transcription factor	CBX3	2	0.00873362	2.89082969	0.14192734	1
14	Transcription factor	CDK	1	0.5	165.5	0.00901573	0.9106
15	Transcription factor	CDX2	1	0.16666667	55.16666667	0.0209417	1
16	Transcription factor	CEBPA	1	0.01449275	4.79710145	0.19495365	1
17	Transcription factor	CEBPB	1	0.0042735	1.41452991	0.54157779	1
18	Transcription factor	CEBPD	1	0.02040816	6.75510204	0.14246035	1
19	Transcription factor	CREB1	1	0.00163399	0.54084967	0.91629616	1
20	Transcription factor	CTCF	1	0.00307692	1.01846154	0.67637737	1
21	Transcription factor	CTCFL	1	0.00632911	2.09493671	0.39993491	1
22	Transcription	CTNNB1	1	0.05555556	18.38888889	0.05607442	1

	factor						
23	Transcription factor	CUX1	1	0.16666667	55.16666667	0.0209417	1
24	Transcription factor	DNMT3A	1	0.05555556	18.38888889	0.05607442	1
25	Transcription factor	DNMT3B	1	0.09090909	30.09090909	0.03569741	1
26	Transcription factor	E2F1	2	0.00584795	1.93567251	0.27691178	1
27	Transcription factor	E2F6	1	0.00310559	1.02795031	0.67247834	1
29	Transcription factor	EBF1	1	0.01111111	3.67777778	0.24743869	1
29	Transcription factor	EED	1	0.125	41.375	0.02686416	1
30	Transcription factor	EGR3	1	0.33333333	110.33333333	0.01200738	1
31	Transcription factor	EP300	2	0.00573066	1.89684814	0.28594371	1
32	Transcription factor	EPAS1	1	0.0625	20.6875	0.0502857	1
33	Transcription factor	ETS1	1	0.0033557	1.11073826	0.64000382	1
34	Transcription factor	EZH2	2	0.02631579	8.71052632	0.01891926	1
35	Transcription factor	FOS	1	0.00574713	1.90229885	0.43215926	1
36	Transcription factor	FOXA2	1	0.01851852	6.12962963	0.15581742	1
37	Transcription factor	FOXM1	1	0.01265823	4.18987342	0.22027756	1
38	Transcription factor	FOXP3	1	0.14285714	47.28571429	0.0239063	1
39	Transcription factor	GATA1	1	0.00471698	1.56132075	0.50348587	1
40	Transcription factor	GATA2	1	0.02	6.62	0.14514435	1
41	Transcription factor	GATA4	1	0.01369863	4.53424658	0.2051561	1
42	Transcription factor	GATA6	1	0.02857143	9.45714286	0.10421666	1
43	Transcription factor	GLI1	1	0.5	165.5	0.00901573	0.9106
44	Transcription factor	HDAC1	1	0.025	8.275	0.11801892	1
45	Transcription factor	HDAC3	1	0.02702703	8.94594595	0.10975686	1
46	Transcription factor	HDAC4	1	0.11111111	36.77777778	0.02981529	1

47	Transcription factor	HIF1A	2	0.00673401	2.22895623	0.22035915	1
48	Transcription factor	HMGA1	1	0.2	66.2	0.01797035	1
49	Transcription factor	HOXD10	1	0.5	165.5	0.00901573	0.9106
50	Transcription factor	IL1B	1	0.33333333	110.33333333	0.01200738	1
51	Transcription factor	IL4	1	0.5	165.5	0.00901573	0.9106
52	Transcription factor	JUN	2	0.01162791	3.84883721	0.08562132	1
53	Transcription factor	JUNB	1	0.09090909	30.09090909	0.03569741	1
54	Transcription factor	JUND	1	0.0044843	1.48430493	0.52281685	1
55	Transcription factor	KDM5B	1	0.00170358	0.56388416	0.90393358	1
56	Transcription factor	KMT2A	2	0.01709402	5.65811966	0.04236922	1
57	Transcription factor	MAX	2	0.00272851	0.90313779	0.76345695	1
58	Transcription factor	MAZ	1	0.00200401	0.66332665	0.84929022	1
59	Transcription factor	MED1	1	0.003367	1.11447811	0.63860033	1
60	Transcription factor	MITF	2	0.03703704	12.25925926	0.00992569	1
61	Transcription factor	MTA3	1	0.0625	20.6875	0.0502857	1
62	Transcription factor	MYC	4	0.00630915	2.08832808	0.05301058	1
63	Transcription factor	NFE2L2	2	0.02777778	9.19444444	0.01708752	1
64	Transcription factor	NFKB1	2	0.01550388	5.13178295	0.05073385	1
65	Transcription factor	NFKB2	1	0.04347826	14.39130435	0.07043045	1
66	Transcription factor	NR1H4	1	0.16666667	55.16666667	0.0209417	1
67	Transcription factor	NR3C1	1	0.00628931	2.08176101	0.40198817	1
68	Transcription factor	NRF1	1	0.00274725	0.90934066	0.72396282	1
69	Transcription factor	PAX5	1	0.00980392	3.24509804	0.27625301	1
70	Transcription factor	PML	1	0.00869565	2.87826087	0.30652624	1
71	Transcription	PPARG	2	0.04651163	15.39534884	0.00645334	0.6518

	factor						
72	Transcription factor	RB1	1	0.04545455	15.04545455	0.06757244	1
73	Transcription factor	RBBP5	1	0.02380952	7.88095238	0.12349493	1
74	Transcription factor	RCOR1	1	0.01754386	5.80701754	0.16375639	1
75	Transcription factor	RELA	2	0.00454545	1.50454545	0.40651385	1
76	Transcription factor	REST	1	0.00452489	1.49773756	0.51934486	1
77	Transcription factor	RFX5	1	0.01010101	3.34343434	0.2691284	1
78	Transcription factor	RUNX3	1	0.00632911	2.09493671	0.39993491	1
79	Transcription factor	SMAD1	1	0.02272727	7.52272727	0.1289454	1
80	Transcription factor	SOX9	1	0.015625	5.171875	0.18206284	1
81	Transcription factor	SP1	2	0.00383142	1.26819923	0.51553093	1
82	Transcription factor	SPI1	1	0.00358423	1.18637993	0.61262861	1
83	Transcription factor	STAT1	2	0.00445434	1.47438753	0.4185747	1
84	Transcription factor	STAT3	3	0.01119403	3.70522388	0.02858138	1
85	Transcription factor	STAT5A	1	0.05882353	19.47058824	0.05318338	1
86	Transcription factor	STAT5B	1	0.07142857	23.64285714	0.04447039	1
87	Transcription factor	SUMO2	1	0.00342466	1.13356164	0.6315212	1
88	Transcription factor	TAF1	1	0.00176991	0.58584071	0.89196447	1
89	Transcription factor	TBL1XR1	1	0.03225806	10.67741935	0.09305872	1
90	Transcription factor	TCF12	1	0.00534759	1.77005348	0.45737112	1
91	Transcription factor	TCF4	1	0.00884956	2.92920354	0.30193181	1
92	Transcription factor	TFAP4	1	0.00465116	1.53953488	0.50881514	1
93	Transcription factor	TGFB1	3	0.09677419	32.03225806	0.00006038	6.10e-3
94	Transcription factor	TLR2	1	0.25	82.75	0.01499225	1
95	Transcription factor	TNFSF12	1	0.14285714	47.28571429	0.0239063	1

96	Transcription factor	TP53	3	0.01863354	6.16770186	0.00673744	0.6805
97	Transcription factor	TRIM25	1	0.01587302	5.25396825	0.17946622	1
98	Transcription factor	TRIM28	2	0.00854701	2.82905983	0.1473263	1
99	Transcription factor	YY1	2	0.0052356	1.73298429	0.32913954	1
100	Transcription factor	ZBTB16	1	0.04761905	15.76190476	0.06470784	1
101	Transcription factor	ZNF143	1	0.00322581	1.06774194	0.65652885	1
