# Supplementary material for: A Group of Tumor-Suppressive micro-RNAs Changes Expression Coordinately in Colon Cancer
Source: Curr Issues Mol Biol. 2023 Jan 20;45(2):975–89. doi: 10.3390/cimb45020063 (PMC9955927; doi:10.3390/cimb45020063)
Supplement: Supplementary file 1 [file cimb-45-00063-s001.zip › Supplementary Table S2-Network modules.pdf]

Table S2- Modules in the transcription factors enrichment network [33]

No	Module	Nodes in module	Node list	Genes in nodes
1	Cardiovascular, muscular, vascular, embryonic differentiation, regulation of epithelial-to-mesenchymal transition	48	aortic valve development,cardiac muscle cell proliferation,cardiac muscle tissue development,cell migration involved in heart development,cell proliferation involved in heart morphogenesis,cell surface receptor signaling pathway involved in heart development,chordate embryonic development,coronary vasculature development,coronary vasculature morphogenesis,embryo development ending in birth or egg hatching,embryonic organ development,endocardial cushion formation,epithelial tube morphogenesis,heart development,heart valve development,heart valve morphogenesis,histone deacetylation,macromolecule deacylation,mesenchymal cell differentiation,mesenchyme morphogenesis,muscle cell differentiation,muscle tissue development,myotube differentiation,negative regulation of extracellular matrix organization,negative regulation of growth,negative regulation of muscle cell differentiation,negative regulation of striated muscle cell differentiation,ossification,positive regulation of cardiac muscle tissue growth,positive regulation of glial cell differentiation,positive regulation of heart growth,positive regulation of organ growth,protein deacetylation,protein deacylation,pulmonary valve development,regulation of blood vessel endothelial cell migration,regulation of cardiac epithelial to mesenchymal transition,regulation of cardiac muscle cell proliferation,regulation of epithelial to mesenchymal transition,regulation of muscle cell differentiation,regulation of myotube differentiation,regulation of striated muscle cell differentiation,semi-lunar valve development,somitogenesis,striated muscle cell differentiation,striated muscle tissue development,vascular associated smooth muscle cell differentiation,vasculogenesis	*NOTCH1,GLI1,TGFβ, PDGF,ATF4,SUFU, CTNNB1,STAT4,CDX1 CDX2,KMT2A,TRIM28 NFATC1,EP300,RCOR1, BRCA1,HDAC3,HDAC4, SMARCA4*
2	Cell cycle- G1-S and G2-M transitions, cell cycle checkpoints	46	DNA damage checkpoint,DNA damage response, signal transduction by p53 class mediator,DNA damage response, signal transduction resulting in transcription,DNA integrity checkpoint,G1 DNA damage checkpoint,G1/S transition of mitotic cell cycle,cell cycle G1/S phase transition,cell cycle G2/M phase transition,cell cycle arrest,cell cycle checkpoint,cyclin-dependent protein kinase holoenzyme complex,intracellular signal transduction involved in G1 DNA damage checkpoint,intrinsic apoptotic signaling pathway,microtubule organizing center organization,mitotic DNA damage checkpoint,mitotic DNA integrity checkpoint,mitotic G1 DNA damage checkpoint,mitotic G1/S transition checkpoint,mitotic cell cycle checkpoint,negative regulation of G1/S transition of mitotic cell cycle,negative	*JUN,MYC,CREB1,FOS, CTNNB1,NFKB1,TGFβ, SMARCA4,RBBP4, BRCA1*

			regulation of cell cycle G1/S phase transition,negative regulation of cell cycle phase transition,negative regulation of mitotic cell cycle,negative regulation of mitotic cell cycle phase transition,positive regulation of cell cycle,positive regulation of cell cycle arrest,positive regulation of cell cycle process,protein kinase complex,protein modification by small protein removal,regulation of G1/S transition of mitotic cell cycle,regulation of cell cycle G1/S phase transition,regulation of cell cycle arrest,regulation of cyclin-dependent protein kinase activity,response to UV,response to light stimulus,serine/threonine protein kinase complex,signal transduction by p53 class mediator,signal transduction in response to DNA damage,signal transduction involved in DNA damage checkpoint,signal transduction involved in DNA integrity checkpoint,signal transduction involved in cell cycle checkpoint,signal transduction involved in mitotic DNA damage checkpoint,signal transduction involved in mitotic DNA integrity checkpoint,signal transduction involved in mitotic G1 DNA damage checkpoint,signal transduction involved in mitotic cell cycle checkpoint,transferase complex, transferring phosphorus-containing groups	
3	DNA transcription	39	DNA-templated transcription, initiation,RNA polymerase II cis-regulatory region sequence-specific DNA binding,RNA polymerase II-specific DNA-binding transcription factor binding,activating transcription factor binding,carbohydrate biosynthetic process,cellular response to steroid hormone stimulus,cellular response to transforming growth factor beta stimulus,euchromatin,foam cell differentiation,hormone receptor binding,hormone-mediated signaling pathway,interaction with symbiont,intracellular receptor signaling pathway,megakaryocyte differentiation,modulation by host of symbiont process,negative regulation of carbohydrate metabolic process,negative regulation of small molecule metabolic process,nuclear chromatin,nuclear hormone receptor binding,nuclear receptor binding,positive regulation of DNA binding,positive regulation of viral transcription,regulation of DNA binding,regulation of DNA-templated transcription, initiation,regulation of binding,regulation of carbohydrate biosynthetic process,regulation of carbohydrate metabolic process,regulation of intracellular steroid hormone receptor signaling pathway,regulation of symbiotic process,regulation of transcription regulatory region DNA binding,regulation of viral process,regulation of viral transcription,response to steroid hormone,response to thyroid hormone,response to transforming growth factor beta,steroid hormone mediated signaling pathway,steroid hormone receptor binding,transmembrane receptor protein serine/threonine kinase signaling pathway,viral transcription	*ELF1,CDK2,E2F1,CCND1, CDK2.MYC,ORC3,CCNE1, CCNA2,BRCA1,EP300, E2F4,TP53,RAD21,ATF4, GLI1,PDGFR1,RBBP4*
4	MiRNA synthesis	35	dsRNA processing,gene silencing,import into nucleus,negative regulation of gene silencing by	*STAT3,TGFβ,CDK2,E2F1, FOS,ETS1,HDAC3,TP53,*

	regulation<br>Intracellular protein transport		RNA,negative regulation of gene silencing by miRNA,negative regulation of posttranscriptional gene silencing,nuclear transport,nucleocytoplasmic transport,positive regulation of cellular protein localization,positive regulation of establishment of protein localization,positive regulation of gene silencing by miRNA,positive regulation of intracellular protein transport,positive regulation of intracellular transport,positive regulation of nucleocytoplasmic transport,positive regulation of posttranscriptional gene silencing,positive regulation of protein import,positive regulation of protein localization to nucleus,positive regulation of protein transport,pri-miRNA transcription by RNA polymerase II,production of miRNAs involved in gene silencing by miRNA,production of small RNA involved in gene silencing by RNA,protein import,protein import into nucleus,protein localization to nucleus,regulation of gene silencing by miRNA,regulation of intracellular protein transport,regulation of intracellular transport,regulation of nucleocytoplasmic transport,regulation of pri-miRNA transcription by RNA polymerase II,regulation of production of miRNAs involved in gene silencing by miRNA,regulation of production of small RNA involved in gene silencing by RNA,regulation of protein import,regulation of protein import into nucleus,regulation of protein localization to nucleus,regulation of reactive oxygen species metabolic process	*EED,TRIM28,BRCA1*
5	Response to stress and different stimuli including through transcription<br>Endoplasmic reticulum stress response (UPR)<br>Apoptosis	35	ER-nucleus signaling pathway,Notch signaling pathway,adaptive thermogenesis,cellular response to abiotic stimulus,cellular response to arsenic-containing substance,cellular response to decreased oxygen levels,cellular response to environmental stimulus,cellular response to ionizing radiation,cellular response to light stimulus,cellular response to oxygen levels,cellular response to radiation,cellular response to topologically incorrect protein,circadian rhythm,cold-induced thermogenesis,negative regulation of cell population proliferation,positive regulation of apoptotic process,positive regulation of transcription from RNA polymerase II promoter in response to stress,positive regulation of transcription from RNA polymerase II promoter involved in cellular response to chemical stimulus,regulation of DNA-templated transcription in response to stress,regulation of Notch signaling pathway,regulation of carbohydrate catabolic process,regulation of cold-induced thermogenesis,regulation of fibroblast proliferation,regulation of transcription from RNA polymerase II promoter in response to stress,response to arsenic-containing substance,response to drug,response to endoplasmic reticulum stress,response to gamma radiation,response to hypoxia,response to ionizing radiation,response to topologically incorrect protein,response to unfolded protein,rhythmic process,stem	*TP53,ATF4,ATF3,HSF1, MYCD,JUN, EP300,HDAC3,KMT2A, NOTCH1,CTNNB1, STAT3,STAT1,NFKB1*

			cell population maintenance,temperature homeostasis	
6	Urinary system development	28	angiogenesis,cell proliferation involved in kidney development,fibroblast proliferation,kidney development,kidney epithelium development,kidney mesenchyme development,maintenance of cell number,mesenchymal cell proliferation,mesenchyme development,metanephros development,muscle cell proliferation,nephron development,nephron epithelium development,nephron tubule development,nephron tubule epithelial cell differentiation,positive regulation of DNA metabolic process,regulation of angiogenesis,regulation of epithelial cell differentiation involved in kidney development,regulation of lipid metabolic process,regulation of mesenchymal cell proliferation,regulation of smooth muscle cell proliferation,regulation of type I interferon production,regulation of vasculature development,renal system development,renal tubule development,smooth muscle cell proliferation,type I interferon production,urogenital system development	*STAT1,NFKB1,CTNNB1, PDGFR,NOTCH1,MYC, E2F2,ETS1,CREB1,EP300, BRCA1,TRIM28*
7	Transcription regulation through DNA methylation, chromatin and chromosomes regulation VEGF production	21	DNA alkylation,DNA methylation or demethylation,centromeric sister chromatid cohesion,histone H3-K4 methylation,histone H3-K9 acetylation,histone lysine methylation,macromolecule methylation,methylation,positive regulation of chromatin organization,positive regulation of chromosome organization,positive regulation of histone methylation,positive regulation of histone modification,regulation of chromatin organization,regulation of chromosome organization,regulation of histone H3-K4 methylation,regulation of histone methylation,regulation of sister chromatid cohesion,regulation of vascular endothelial growth factor production,response to alcohol,sister chromatid cohesion,vascular endothelial growth factor production	*CTNNB1,MYC,FOS, KMT2A,BRCA1,RAD21, TRIM28,CTCF,ATF4*
8	Hematopoesis	13	cellular response to interleukin-6,erythrocyte differentiation,erythrocyte homeostasis,homeostasis of number of cells,myeloid cell differentiation,myeloid cell homeostasis,positive regulation of defense response,positive regulation of myeloid cell differentiation,positive regulation of transcription by RNA polymerase II,regulation of erythrocyte differentiation,regulation of hemopoiesis,regulation of myeloid cell differentiation,response to interleukin-6	*STAT1,STAT3,CEBPA, ETS1,MED1,KMT2A, SMARCA4*
9	Response to different chemical or physical stress	9	Fc receptor signaling pathway,RNA polymerase II transcription regulator complex,SMAD binding,cellular response to chemical stress,cellular response to inorganic substance,cellular response to oxidative stress,response to cadmium ion,response to oxidative stress,transcription regulator complex	*NFKB1,CTNNB1,JUN, FOS,E2F1,CREB1,CDK2, NFATC1,HSF1, TP53,ATF4,CEBPA*
10	Eye development	7	camera-type eye development,cellular response to interleukin-7,eye development,response to interleukin-	*MED4,PDGFR,SMARCA4, ATF4,STAT3*

			7,sensory organ development,sensory system development,visual system development	
11	Chromatin regulation	5	DNA packaging,chromatin DNA binding,chromatin assembly or disassembly,chromatin binding,protein-DNA complex subunit organization	*TRIM28,RBBP4,CTCF, TP53, SMARCA4, EP300, MYC,STAT3,STAT1,E2F4*
12	Cell cycle-G0-G1 transition	4	G0 to G1 transition,histone methyltransferase complex,regulation of G0 to G1 transition,regulation of gene expression, epigenetic	*E2F1,BRCA1,RBBP4,EED, KMT2A*
13	Transcription regulation	3	RNA polymerase II transcription cofactor binding,transcription cofactor binding,transcription corepressor binding	*THRB,HDAC3,EED*
14	Protein sumoxylation	2	protein sumoylation,regulation of protein sumoylation	*HDAC4,CTNNB1*

MiRNA- micro-RNA; TF- transcription factor; G1,S,G2,M-stages in the cell cycle.
