# Supplementary figures and images for: A Group of Tumor-Suppressive micro-RNAs Changes Expression Coordinately in Colon Cancer
Source: Curr Issues Mol Biol. 2023 Jan 20;45(2):975–89. doi: 10.3390/cimb45020063 (PMC9955927; doi:10.3390/cimb45020063)

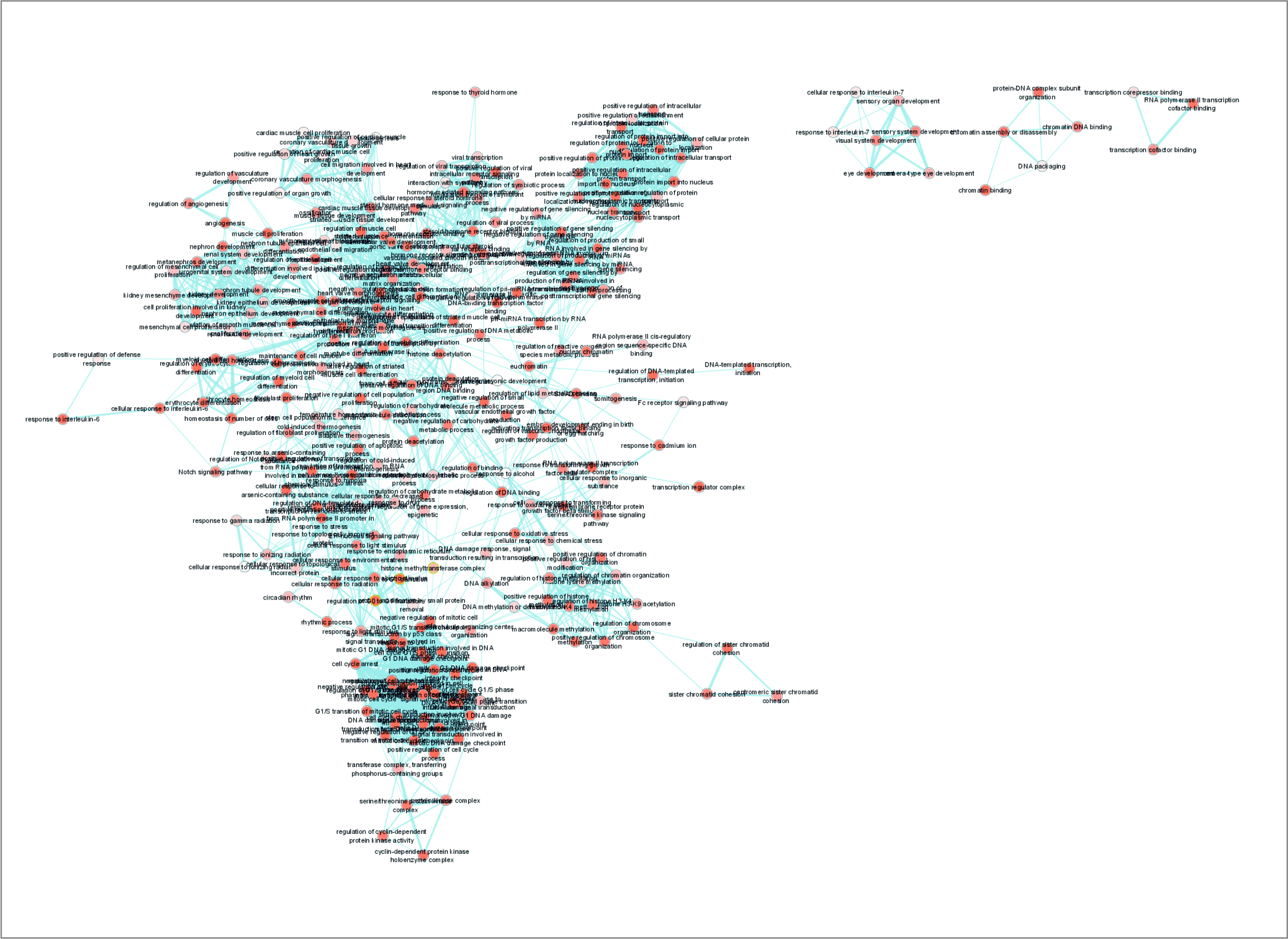

Supplement: Supplementary file 1 [file cimb-45-00063-s001.zip › Figure S1- Enrichment map with annotations.tif]
